# BRAF mutations in thyroid tumors from an ethnically diverse group

**DOI:** 10.1186/1897-4287-10-10

**Published:** 2012-08-27

**Authors:** Hans-Juergen Schulten, Sherine Salama, Zuhoor Al-Mansouri, Reem Alotibi, Khalid Al-Ghamdi, Osman Abdel Al-Hamour, Hassan Sayadi, Hosam Al-Aradati, Adel Al-Johari, Etimad Huwait, Mamdooh Gari, Mohammed Hussain Al-Qahtani, Jaudah Al-Maghrabi

**Affiliations:** 1 Center of Excellence in Genomic Medicine Research, King Abdulaziz University, Jeddah, Saudi Arabia; 2 Department of Pathology, Faculty of Medicine, King Abdulaziz University, Jeddah, Saudi Arabia; 3 Department of Pathology, King Faisal Specialist Hospital and Research Center, Jeddah, Saudi Arabia; 4 Department of Biochemistry, King Abdulaziz University, Jeddah, Saudi Arabia; 5 Department of Surgery, Faculty of Medicine, King Abdulaziz University, Jeddah, Saudi Arabia; 6 Department of Surgery, King Faisal Specialist Hospital and Research Center, Jeddah, Saudi Arabia

**Keywords:** BRAF mutations, Deletion codon 599, Duplication codon 599, Thyroid carcinoma, Follicular adenoma, Family history, MENA region

## Abstract

**Background:**

The molecular etiology of thyroid carcinoma (TC) and other thyroid diseases which may present malignant precursor lesions is not fully explored yet. The purpose of this study was to estimate frequency, type and clinicopathological value of BRAF exon 15 mutations in different types of cancerous and non-cancerous thyroid lesions originating in an ethnically diverse population.

**Methods:**

BRAF exon 15 was sequenced in 381 cases of thyroid lesions including Hashimoto´s thyroiditis, nodular goiters, hyperplastic nodules, follicular adenomas (FA), papillary TC (PTC), follicular variant PTC (FVPTC), microcarcinomas of PTC (micro PTC; tumor size ≤ 1 cm), follicular TC (FTC), and non-well differentiated TC (non-WDTC).

**Results:**

We identified BRAF mutations in one of 69 FA, 72 of 115 (63%) PTC, seven of 42 (17%) FVPTC, 10 of 56 (18%) micro PTC, one of 17 (6%) FTC, and one of eight (13%) non-WDTC. Most of the cases showed the common V600E mutation. One case each of PTC, FVPTC, and FTC harbored a K601E mutation. A novel BRAF mutation was identified in a FA leading to deletion of threonine at codon 599 (p.T599del). A rare 3-base pair insertion was detected in a stage III PTC resulting in duplication of threonine at codon 599 (p.T599dup). Patients with PTC harboring no BRAF mutation (BRAF^wt^) were on average younger than those with a BRAF mutation (BRAF^mut^) in the PTC (36.6 years vs. 43.8 years). Older age (≥ 45 years) in patients with PTC was significantly associated with tumor size ≥ 4 cm (P = 0.018), vessel invasion (P = 0.004), and distant metastasis (P = 0.001). Lymph node (LN) involvement in PTC significantly correlated with tumor size (P = 0.044), and vessel invasion (P = 0.013). Of notice, taken the whole TC group, family history of thyroid disease positively correlated with capsular invasion (P = 0.025).

**Conclusions:**

Older age is manifold associated with unfavorable tumor markers in our series. The K601E identified in a PTC, FVPTC, and FTC seems to be more distributed among different histological types of TC than previously thought. The T599del is a yet undescribed mutation and the rare T599dup has not been reported as a mutation in PTC so far.

## Background

TC represents a considerable cancer burden worldwide [1,2]. In Saudi Arabia it is considered as the fourth most common cancer type with 6.4% of all diagnosed cancers [3]. Median age of women diagnosed with TC in Saudi Arabia is considerable lower than for men (37 years *vs.* 43 years). Notably, females in this country are more commonly diagnosed with FVPTC (19.3% *vs.* 9.6%) and micro PTC (7.4% *vs.* 2.6%) than males.

Differentiated types of TC arising from endodermally derived follicular cells comprise papillary TC (PTC), follicular TC (FTC), and Hurthle cell carcinoma. Hurthle cell carcinomas are also considered as a subtype of FTC. Anaplastic TC (ATC) is considered as undifferentiated TC which either may evolve *de novo* or from PTC or FTC [4]. Medullary TC (MTC) are derived from calcitonin-producing C cells and own a different etiology involving commonly mutations in the RET oncogene. PTC represents about 80% of differentiated TC with an increasing trend while FTC (~11%), Hurthle cell carcinoma (~3%), MTC (~4%), and ATC (~2%) represent minor variants [2]. A study on the pattern of TC arising in Jeddah, an ethnically diverse metropolis in Western Saudi Arabia, revealed a common distribution of histological types: PTC (82%), FTC (4.4%), ATC (6.7%), and MTC (6.7%) [5].

The pathogenesis of the majority of TC is affected by somatic mutations or rearrangements in known TC genes. The BRAF gene is the most frequently mutated gene in TC. BRAF is a cytoplasm receptor serine/threonine kinase and a key molecule in the mitogen activated protein kinase (MAPK) pathway. It is mutated in diverse human malignancies although the frequency varies significantly between different types of cancers [6]. In malignant melanomas and in TC, which both are oncogenic transformations of neural crest derived cell lineages, frequency of BRAF mutations can exceed 80% of the cases [6,7]. BRAF activation affects in the vast majority of cases codon 600 located within the regulatory domain encoded by exon 15. Most common mutation at codon 600 is a valine-to-glutamic acid transversion (V600E). This mutation disrupts the inactive conformation of BRAF as it mimics activational phosphorylation at the adjacent residues and results in constitutive downstream signaling [8]. Other BRAF activation mutations are far less common in TC like the K601E mutation that is commonly restricted to FVPTC [9]. Although the prognostic impact of BRAF mutations in PTC is controversially discussed, the V600E mutation seems to be a valid target for molecular therapy as this mutation confers elevated resistance to iodine ablation [7,10]. We assessed the impact of BRAF mutations in cancerous and non-cancerous thyroid lesions originating in an ethnically diverse population by a comprehensive mutational survey.

## Methods

### Thyroid disease cases

We examined 381 cases of thyroid lesions from 376 patients which were treated surgically in the period between January 1995 to June 2011 at the King Abdulaziz University Hospital, Jeddah, and the King Faisal Specialist Hospital & Research Center, Jeddah, or were referral/consultant cases from other regional hospitals. In five cases, tumor and non-tumor lesions from the same patient were evaluated in the study separately. Saudi Arabian nationality was reported for 61% of the patients and 27% originated from other MENA (Middle East and North Africa) countries. A minority of patients originated from other world regions (8%) or patient´s nationality was not recorded (4%). Histopathological diagnosis and staging of thyroid lesions was performed by an oncologic pathologist (JM) according to established criteria [11,12]. Cases were selected on basis of sample and data availability. With the exception of MTC [13] the most common, surgically treated thyroid diseases were included in the study, i.e. Hashimoto´s thyroiditis, nodular goiters, hyperplastic lesions, FA, FTC (including Hurthle cell carcinomas), PTC (> 1 cm), FVPTC (> 1 cm), micro PTC (≤ 1 cm) and non-well differentiated TC (non-WDTC), The non-WDTC group comprised poorly differentiated insular variant PTC, combinatorial PTC/ATC, and ATC. PTC (> 1 cm) and FVPTC (> 1 cm) are hereinafter referred as PTC and FVPTC, respectively.

Demographic and clinicopathological data were compiled from patients’ files and included age, gender, family history of thyroid disorders/lesions, histological type, vessel (lymphatic, perineural, or vascular) invasion, capsular invasion, tumor extension, tumor size, multifocal tumors (including cases of subsequent partial thyroidectomies), lymph node involvement, distant metastasis (comprising radiologic findings), and tumor stage. Information to family history of thyroid disorders/lesions was voluntarily given by patients and regarded as positive if at least one first degree relative or two second degree relatives were affected. This study was approved by the ethical review boards of both institutions.

### Mutational screening

Included in mutational analysis of BRAF exon 15 were specimens from primary thyroid lesions except in 15 cases in which only specimens from a recurrence or metastasis were available. A pathologist (JM) has reviewed each case and chose only specimens with not less than 70% of abnormal or tumor cells, respectively. Genomic DNA was extracted in the majority of cases from 10 μm sections of formalin-fixed and paraffin-embedded (FFPE) material and using conventional xylene/ethanol treatment, overnight incubation with proteinase K, and subsequent DNA purification utilizing the QIAmp DNA FFPE tissue kit (Qiagen, Hilden, Germany). In 116 cases native or fresh-frozen (FF) specimens were preserved and in these cases the QiAmp DNA mini kit was used for DNA purification. DNA concentration was measured with the Nanodrop device (Thermo Scientific, Wilmington, DE).

The standard PCR protocol was performed as described earlier [13]. PCR products were checked by electrophoresis on 2% agarose gels. Purified PCR products were subjected to cycle sequence reactions using nested primers overlapping with the PCR primers and the BigDye Terminator V3.1 Cycle Sequencing kit (Applied Biosystems, Foster City, CA, USA). Purified sequencing products were finally resolved by capillary electrophoresis on an ABI PRISM 3130 Sequencer. Sequences were screened for BRAF exon 15 mutations using a combination of manual readout of electropherograms and the online NCBI's BLAST database [14].

### Statistical analysis

Associations of demographic data, clinicopathological factors, and BRAF mutational status (BRAF^wt^*vs.* BRAF^mut^) in histological types of TC were evaluated using the non-parametric Wilcoxon rank sum test or Fisher´s exact test for contingency tables in the 2-sided configuration. P-value for accepting significance was p = 0.05. Statistical analysis was performed using the SPSS statistics 16 program (IBM Corp., New York, NY).

## Results

The hotspot region in BRAF exon 15 was screened for mutations in 381 cases of cancerous and non-cancerous thyroid lesions. The case series comprised 10 Hashimoto´s thyroiditis lesions, 46 nodular goiters, 18 hyperplastic nodules, 69 FA, 115 PTC, 42 FVPTC, 56 micro PTC (≤ 1 cm), 17 FTC, and eight non-WDTC (2 insular PTC, 1 PTC/ATC, and 5 ATC) (Table 1). Mean age at diagnosis in non-malignant lesions ranged between 34.4 years in patients with hyperplastic nodules and 41.9 years in patients with goiter. In the TC group, mean age varied between 36.4 years in patients with micro PTC and 59 years in non-WDTC cases. Patients with micro PTC were on average 4 years younger than their PTC and FVPTC counterparts. Number of female cases predominated in all histological types. Of notice, female to male ratio was considerably higher in micro PTC than in PTC (6:1 *vs.* 2.3:1). Almost all micro PTC were classified as stage I tumors except two multifocal stage III tumors (follicular variant in one case) and one stage IV tumor (oncocytic variant) (Table 2). The majority of cases were stage I tumors in PTC (64%), FVPTC (57%), and FTC (65%). Seven of eight non-WDTC cases were stage IV tumors and one was a stage II tumor. Follow-up period in TC patients ranged between 0 and 23 years (mean, 3.2 years; SD 3.2). Distant metastases were reported for PTC (15%), FVPTC (17%), FTC (6%), and non-WDTC (50%). Eight patients deceased from disease (age at first diagnosis, 49 to 78 years; mean age, 61 years); tumor progression in these patients was primarily confined to regional sites in two cases (1 PTC and 1 PTC with tall cell features metastasized as ATC), to distant sites in four cases (1 PTC, 1 FTC, 1 PTC/ATC and 1 ATC) and to regional and distant sites in two cases (1 PTC and 1 FVPTC). The mean survival period of these patients was 3.9 years (SD 3.4).

**Table 1:** Demographic and BRAF mutational survey in thyroid lesions

**Thyroid lesion**	**Number**	**Age at diagnosis (years, SD)**	**Female/male ratio**	**Family history**		**BRAF status**^**1**^	
				**neg**	**pos**	**wt**	**mut**
Thyroiditis	10	36.7 ±12.5	10:0	5	2	10	0
Goiter	46	41.9 ±11.4	2.3:1	19	5	46	0
Hyperplastic	18	34.4 ±12.5	8:1	8	0	18	0
FA	69	38.4 ±13.1	3:1	11	2	68	1 T599del
FTC	17	40.4 ±16.7	3.3:1	2	2	16	1 K601E
PTC	115	41.1 ±15.5	2.3:1	27	4	43	1 T599insT, 70 V600E, 1 K601E
FVPTC	42	40.5 ±15.6	5:1	9	4	35	6 V600E, 1 K601E
Micro PTC	56	36.4 ±13.5	6:1	10	4	46	10 V600E
Non-WDTC	8	59.0 ±10.1	7:1	1	1	7	1 V600E

^1^Wt, BRAF wild type; mut, BRAF mutated.

**Table 2:** Distribution of tumor stages and BRAF mutations in thyroid malignancies

**Stage**	**PTC**	**FVPTC**	**micro PTC**	**FTC**	**non-WDTC**
I	74 [31/43]	24 [19/5]	53 [45/9]	11 [10/1]	0
II	9 [4/5]	6 [4/2]	0	2	1 [0/1]
III	15 [3/12]	7	2 [1/1]	3	0
IV	15 [5/10]	4	1	1	7
Unknown	2 [0/2]	1			

BRAF^wt^/BRAF^mut^ cases in brackets.

**Figure 1:**
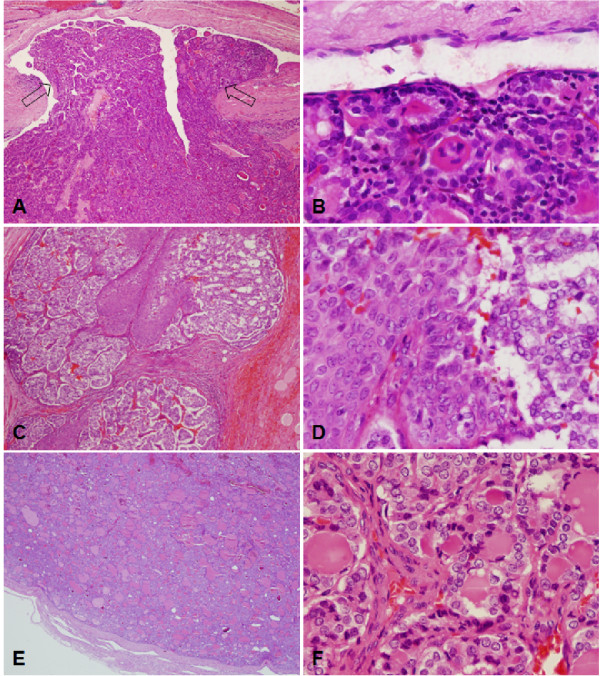
**Hematoxylin and eosin stains of thyroid cancer cases harboring new or rare BRAF mutations.** Left column, original magnification 40 x; right column, original magnification 400 x. **A** and **B**, FTC with a BRAF K601E mutation. Case has a single minimally invasive focus, marked by arrows. **C** and **D**, PTC with BRAF K601E mutation. The recurrence of the case involves subcutaneous tissue and skeletal muscle. Conventional PTC and squamous components are intermixed. **E** and **F**, FA harboring a deletion of BRAF codon 599. The tumor presents as a solitary encapsulated nodule and misses nuclear features of PTC.

**Figure 2:**
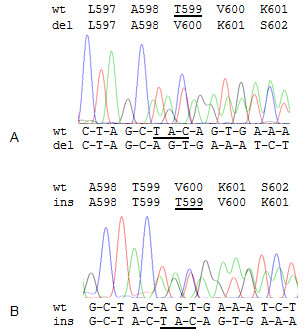
**New and sporadic BRAF mutations in thyroid neoplasms. A**, a new 3-base-pair deletion at codon 599 identified in a FA resulting in deletion of threonine 599 (p.T599del). **B**, a rare 3-base pair insertion at codon 599 found in an aggressive PTC resulting in duplication of threonine 599 (p.T599dup). Deleted and inserted nucleotides and codons are underlined.

Among non-malignant thyroid lesions one of 69 FA exhibited a BRAF mutation. In thyroid malignancies we identified BRAF mutations in 72 of 115 PTC (63%) seven of 42 (17%) FVPTC, 10 of 56 (18%) micro PTC, one of 17 (6%) FTC, and one of eight (13%) non-WDTC (Table 1). Most of the BRAF mutations were the common point mutation in codon 600 leading to substitution of valine by glutamic acid (V600E). One PTC, FVPTC, and FTC each harbored a point mutation in codon 601 resulting in substitution of lysine by glutamic acid (K601E). The K601E mutation that is usually associated with FVPTC histology was identified in case of the FTC (Figure 1A, B) in FF as well as FFPE samples of the tumor. The K601E mutation in the PTC was detected in the primary tumor as well as in the regional progressive metastasis (Figure 1C, D). A yet undescribed BRAF mutation was detected in different sections of a FA (Figure 1E, F). This 3-base-pair deletion at codon 599 erases threonine 599 (T599del) (Figure 2A). A very rare BRAF 3-base-pair insertion at codon 599, resulting in duplication of threonine 599 (T599dup), was detected in a multifocal, stage III PTC that developed distant metastases within 4.5 years (Figure 2B). Both, T599del and T599dup were confined to the tumor.

Tumor size ≥ 4 cm (P = 0.018), vessel invasion (P = 0.004), and distant metastasis (P = 0.001) were significantly more common in PTC patients aged 45 years and older (Table 3). Patients with BRAF^wt^ PTC were on average younger than those with BRAF^mut^ PTC (36.6 years *vs.* 43.8 years; P = 0.025). LN involvement in PTC associated with tumor size (P = 0.044), and vessel invasion (P = 0.013). Tumor size was slightly, but not significantly, larger in BRAF^mut^ than in BRAF^wt^ PTC (3.6 cm *vs.* 2.9 cm; determined for 87 cases). Considering all histological types of TC as a group, i.e. PTC, FVPTC, micro PTC, FTC, and non-WDTC, family history of thyroid disease positively correlated with capsular invasion (P = 0.025).

**Table 3:** Significant associations of demographic/clinocopathological data and BRAF mutations in thyroid malignancies

**Factors**^**1**^	**Histology**	**P value**	**Test**^**2**^
Age ≥ 45 years *vs.* tumor size ≥ 4 cm [87]	PTC	0.018	FET
Age ≥ 45 years *vs.* vessel invasion [113]	PTC	0.004	FET
Age ≥ 45 years *vs.* distant metastasis [115]	PTC	0.001	FET
LN + *vs.* tumor size ≥ 4 cm [43]	PTC	0.044	FET
LN + *vs.* vessel invasion [59]	PTC	0.013	FET
Increasing age *vs.* BRAF^mut^ [115]	PTC	0.025	MWU
Family history *vs.* capsular invasion [64]	TC	0.025	FET

^1^Number of cases included in analysis in brackets; ^2^FET, Fisher´s exact test; MWU, Mann–Whitney U test.

## Discussion

This study is one of the first comprehensive surveys on BRAF mutations in different types of thyroid diseases carried out so far in the MENA region. In general, frequency, distribution, and risk assessment of BRAF mutations in TC differ between studies emphasizing that the genetic etiology of TC is complex and varies between the populations studied [15,16]. The overall incidence of BRAF mutations identified in our series and the gradation of frequency of BRAF mutations between PTC and FVPTC are similar to those displayed in other studies [17]. The detection rate of BRAF^mut^ in PTC varies between approximately 30% and 85% while for FVPTC the rate varies between 0% and 35% with on average ~15% measured across different studies [7,17-20]. Only a few studies have revealed a similar large discrepancy in age distribution between BRAF^wt^ and BRAF^mut^ tumors as we did [21-23] whereas other investigators did not reveal any significant difference and a metanalysis on the clinicopathological impact of BRAF mutations in PTC could not compile age specific differences across six evaluated studies [17,24]. The overall young age distribution in our series can be attributed to the young population structure of the region.

In general, significance of the BRAF mutational status in regard to clinicopathological features and clinical treatment of the disease is still matter of debate although a trend of BRAF mutation with aggressive tumor markers like vessel invasion, capsular invasion, tumor extension, or LN metastases has been revealed in a number of studies [10,16,17,24,25]. We attribute the low clinicopathological significance of BRAF mutations in our series to the fact that this driver mutation is already present in the majority of low stage PTC. The specific correlation of BRAF mutations with poor outcome seems to become more evident in long-term follow-up studies [23,26]. In contrast, BRAF mutations in micro PTC are less likely to correlate with clinicopathological factors [27]. These data coincide with our findings. Our observation that capsular invasion correlates with family history of TC warrants further exploration to sustain these findings and to identify possible genetic predisposition effects in affected individuals.

The K601E mutation, identified in our series in one case each of FTC, FVFTC, and PTC is known to be associated with FVPTC histology [9,24,28-30]. BRAF mutations in TC others than the V600E mutation are commonly associated with non-aggressive tumors [31]; however, association of T599dup and K601E mutations with advanced TC has been reported [32,33].

BRAF mutations in FA have only been identified so far sporadically and are almost K601E mutations which FA have in common with their follicular malignant counterparts [30,34,35]. The novel T599del mutation detected in a FA in our series affects one of the two critical phosphorylation sites (T599 and S602) in the activation domain encoded by exon 15. Phosphorylation of these residues disrupts the hydrophobic association of the phosphate-binding loop (P loop) with the activation loop (A loop) [8,36]. It can be hypothesized that deletion of codon 599 confers a conformational change in a similar manner as phosphorylation of T599 which results in destabilization of the inactive conformation and switch into the active configuration. Further studies have to reveal if the T599del is functionally linked to FA development which would separate it from other BRAF mutations evolving in TC.

The T599dup identified in an aggressive PTC in our series has been recently uncovered as an activation mutation in pilocytic astrocytomas and a small number of ATC with tall cell features [33,37,38]. The T599dup exhibited an elevated kinase activity in transfection assays similarly to V600E [38]. The authors supposed that addition of an extra amino acid residue at this position rather than addition of a second threonine phosphorylation site results in disruption of the inhibitory conformation of the activation loop. Of notice, a considerable number of sporadic mutations in BRAF exon 15 affects or co-affects codon 599 in TC [24,39-43]. These mutations tested so far exhibit elevated kinase activity emphasizing the critical cofunction of codon 599 in the BRAF activation process [8,38,40,42].

## Conclusions

Significant findings of our study include new and rare BRAF mutations in malignant and non-malignant thyroid tumors that in cases of T599dup and K601E seem to confer a certain risk for progressive TC. Other notable findings include correlation of capsular invasion with family history of TC whereas BRAF mutational status disclosed only limited associations with clinicopathological factors in our series.

## Abbreviations

ATC, Anaplastic TC; BRAFmut, BRAF mutation; BRAFwt, BRAF wild type; FA, Follicular adenoma(s); FF, Fresh-frozen; FFPE, Formalin-fixed and paraffin-embedded; FTC, Follicular TC; FVPTC, Follicular variant PTC; LN, Lymph node; MAPK, Mitogen activated protein kinase; MENA, Middle East and North Africa; micro PTC, Microcarcinoma of PTC; MTC, Medullary TC; non-WDTC, Non-well differentiated TC; PTC, Papillary TC; TC, Thyroid carcinoma(s).

## Competing interests

The authors declare that they have no competing interests.

## Authors’ contributions

SS, EH, MG, and ZM made substantial contributions to the conception and design of the study. RA performed sequence analyses of PTC. KG, OAH, and AA were responsible for surgeries, oversight of clinical databases and contributed to the conception and design of the study. SS, HS, HA, and JM performed histological examinations. HJS performed statistical analysis and had general oversight of the study. HJS, JM, and MQ interpreted data and drafted the manuscript. All authors read and approved the final manuscript.
